# Supplementary material for: A Likelihood Approach for Real-Time Calibration of Stochastic Compartmental Epidemic Models
Source: PLoS Comput Biol. 2017 Jan 17;13(1):e1005257. doi: 10.1371/journal.pcbi.1005257 (PMC5240920; doi:10.1371/journal.pcbi.1005257)
Supplement: S1 File — (TAR.GZ) [file pcbi.1005257.s014.tar.gz › HSPH_Online-SI-Revision/PF08/n10-extreme/n10-extreme_PF_summary.pdf]

peak -8	R0	$1/\Theta_2$	inf	inflog	newly	newlylog	Reff	AttackRate
mean	28.4	33.	1068.3	0.7	550.2	0.5	30.8	18.6
median	28.6	27.2	273.2	0.5	196.2	0.4	32.1	18.4
Quantile .1	20.7	10.8	108.	0.3	57.1	0.2	22.8	14.3
Quantile .9	35.8	56.3	2807.6	1.3	1206.4	0.9	39.2	23.3
23	36	29						
length	50							

peak -4	R0	$1/\Theta_2$	inf	inflog	newly	newlylog	Reff	AttackRate
mean	26.5	38.1	33.7	0.2	44.6	0.3	26.	13.2
median	28.1	29.1	33.3	0.2	45.2	0.3	27.6	13.3
Quantile .1	13.5	11.5	23.4	0.1	31.3	0.2	13.3	8.5
Quantile .9	34.6	72.7	41.6	0.2	56.1	0.4	34.3	17.3
10	27	31						
length	50							

peak	R0	$1/\Theta_2$	inf	inflog	newly	newlylog	Reff	AttackRate
mean	13.4	44.7	32.2	0.1	15.5	0.1	11.1	15.8
median	12.1	34.9	22.	0.1	12.9	0.1	8.6	14.8
Quantile .1	3.3	9.	7.3	0.	4.4	0.	4.8	6.4
Quantile .9	23.	88.7	64.	0.2	29.4	0.2	22.7	26.3
11	4	1						
length	50							

peak +4	R0	$1/\Theta_2$	inf	inflog	newly	newlylog	Reff	AttackRate
mean	13.2	44.8	95.8	0.2	37.5	0.1	24.1	17.2
median	11.4	35.6	62.4	0.2	19.8	0.1	20.2	15.8
Quantile .1	3.4	9.1	6.7	0.	6.	0.	7.6	7.8
Quantile .9	23.5	87.3	202.1	0.5	77.1	0.2	42.8	29.
12	3	0						
length	50							

peak +8	R0	$1/\Theta_2$	inf	inflog	newly	newlylog	Reff	AttackRate
mean	13.2	42.2	258.4	0.4	140.7	0.3	26.	17.1
median	11.6	31.7	113.6	0.3	71.1	0.2	19.4	16.5
Quantile .1	4.1	8.9	30.9	0.1	18.	0.1	6.1	7.6
Quantile .9	22.9	84.9	493.8	0.8	257.9	0.6	50.6	25.2
14	5	0						
length	50							
